# The emerging role of cellular senescence in amyotrophic lateral sclerosis

**DOI:** 10.3389/fnins.2025.1599492

**Published:** 2025-08-01

**Authors:** Xingli Tan, Naiyong Gao

**Affiliations:** ^1^School of Basic Medicine Sciences, Shandong Second Medical University, Weifang, China; ^2^Department of Neurology, Linyi People’s Hospital, Linyi, Shandong, China

**Keywords:** cellular senescence, amyotrophic lateral sclerosis, aging, anti-senescence, aging of motor neurons

## Abstract

Cellular senescence is a state of permanent cell cycle arrest and is considered a key contributor to aging and age-related diseases, including amyotrophic lateral sclerosis (ALS). The physiological processes of aging lead to a variety of molecular and cellular phenotypes, and evidence of overlap between ALS and aging-related biomarkers suggests that cell type-specific senescence may be a critical factor in ALS. Senescent microglial cells, astrocytes, and neurons have been detected in ALS patients and animal models. However, while accumulating evidence suggests a potential link between cellular senescence and ALS, this connection remains not yet conclusively established. Importantly, how senescent cells may contribute to the neuropathophysiology of ALS remains largely unknown. Additionally, the growing popularity of anti-aging therapies has highlighted the potential of senescent cell clearance as a promising strategy for treating age-related diseases, including ALS. This review provides an overview of cellular senescence, discusses recent advances in understanding how senescence in different cell types influences ALS pathogenesis, and explores the potential role of anti-senescence therapies in ALS treatment.

## 1 Introduction

Amyotrophic lateral sclerosis (ALS) is a common neurodegenerative disorder marked by the progressive degeneration of upper and lower motor neurons. It primarily leads to progressive muscle weakness and atrophy, eventually leading to respiratory failure and death within 3 to 5 years (Meyer, 2021; Irwin et al., 2024). Approximately 90% of ALS cases are classified as sporadic ALS (sALS), with the remaining 10% categorized as familial ALS (fALS; Gois et al., 2020). More than 50 genes have been identified as potential contributors to the pathogenesis or modification of ALS, with SOD1, C9orf72, FUS, and TARDBP being the most frequently implicated (Masrori and Van Damme, 2020; Yun and Ha, 2020). The pathogenesis of ALS is complex and currently implicated in excitotoxicity, nucleocytoplasmic transport defects, impaired protein homeostasis, altered RNA metabolism, DNA repair damage, mitochondrial dysfunction, neuroinflammation, axonal transport deficits, and oligodendrocyte dysfunction (Mejzini et al., 2019).

Cellular senescence is defined at the cellular level by a permanent cell cycle arrest, accompanied by alterations in cell shape, epigenetic modifications and secretory phenotype (Csekes and Rackova, 2021; Gorgoulis et al., 2019). Cellular senescence encompasses both replicative senescence (RS) and stress-induced premature senescence (SIPS). RS refers to cellular senescence primarily induced by telomere shortening and dysfunction under physiological conditions (Aravinthan, 2015; Cristofalo et al., 2004; Daios et al., 2022). SIPS refers to cellular senescence triggered by various stress stimuli, such as oxidative stress (Deng et al., 2023), direct and persistent DNA damage (Ou and Schumacher, 2018), activation of tumor suppressors (Liu et al., 2023) and/or oncogenes (Zhu et al., 2020), mitochondrial dysfunction (Miwa et al., 2022), epigenetic dynamics (Zhu et al., 2021), and therapy-induced senescence (TIS; Prasanna et al., 2021). Cellular senescence may arise at various life stages, ranging from embryonic development to adulthood, but it is predominantly linked to the aging process (Gorgoulis et al., 2019; Rhinn et al., 2019). Cellular senescence is a highly regulated process that plays a crucial role in tumor suppression, aging, wound healing, and embryonic development (Calcinotto et al., 2019). Previous studies have confirmed that cell death is a key consequence of cellular senescence (Ogrodnik, 2021). Cellular senescence has beneficial effects, for example, by preventing tumorigenesis through irreversible cell cycle arrest. However, growing evidence suggests that senescent cells accumulate in aging tissues and organs, disrupting normal physiological functions and playing a role in organismal aging and age-related diseases (Hohn et al., 2017; Tacutu et al., 2011; Baker et al., 2011; Gerenu et al., 2017; Sturmlechner et al., 2017).

As a stress response, cellular senescence is associated with degenerative pathologies of aging. This progressive degeneration occurs at the molecular, cellular, tissue, and organ levels (Regulski, 2017). Senescent cells usually display the following key characteristics: loss of proliferative or regenerative capacity, alterations in metabolic function, and resistance to apoptosis (Khosla et al., 2020).

**Figure 1:**
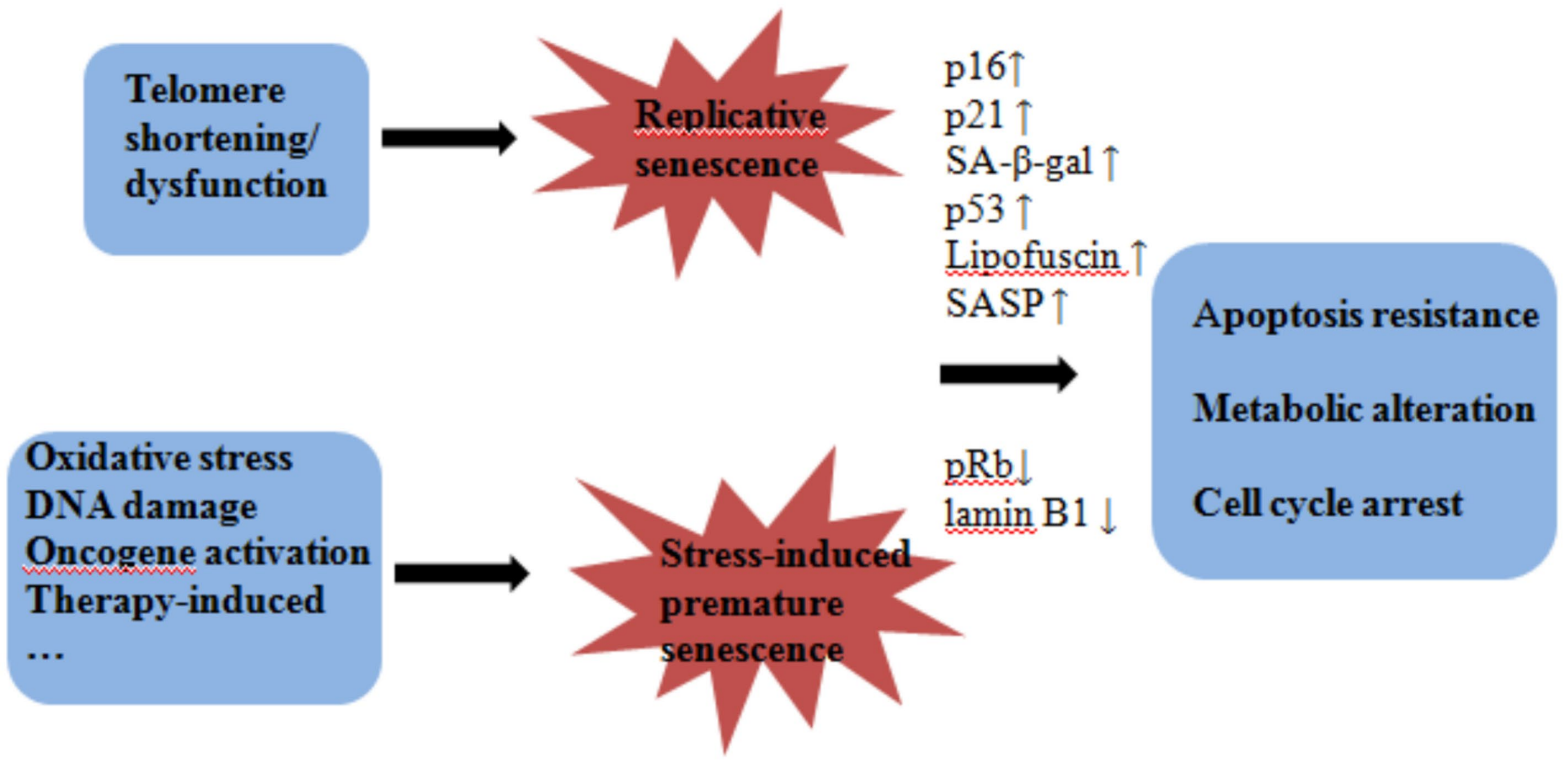
The cause and outcome of cell senescence Telomere shortening/dysfunction induced replicative senescence, while oxidative stress, DNA damage, oncogene activation, and chemotherapy-induced stress led to stress-induced premature senescence. Senescent cells showed increased p16, p21, SA-*β*-gal, and SASP secretion phenotypes, which ultimately led to cell cycle arrest, resistance to apoptosis, and metabolic alterations.

Although the hallmarks of senescent cells have not yet been precisely defined, there is a general consensus on certain key characteristics of senescent cells. Senescent cells become enlarged and flattened morphologically (Khosla et al., 2020; Hernandez-Segura et al., 2018). At the transcriptional level, p16 and p21 are among the most frequently utilized indicators of cellular senescence (Gorgoulis et al., 2019; Gasek et al., 2021). Other markers for identifying senescent cells include elevated activity of senescence-associated *β*-galactosidase (SA-β-gal), elevated expression of p53, decreased phosphorylation of retinoblastoma protein (pRb), accumulation of lipofuscin in the lysosomes, an increase in senescence-associated DNA damage foci (SDFs), and various nuclear changes, such as the loss of lamin B1 (Figure 1). In addition, senescent cells secrete a characteristic pro-inflammatory cytokine profile known as the senescence-associated secretory phenotype (SASP; Gorgoulis et al., 2019). These characteristics are commonly employed to recognize senescent cells in diverse tissues linked to aging or pathological states. Nonetheless, they are not universally present in all senescent cells and may be inadequate for identifying senescent cells *in vivo* (Cohn et al., 2023). Therefore, it is recommended to use multiple markers, particularly in vivo, to identify senescent cells.

Although cellular senescence is recognized as a key contributor to age-associated diseases (Baker et al., 2011; Ogrodnik et al., 2021), different factors may induce senescence in distinct cell types under different pathological circumstances (Khan et al., 2017). The age of onset of ALS typically ranges from 50 to 75 years. Owing to the growing aging population, the number of ALS patients is rapidly increasing, with an estimated 400,000 individuals worldwide expected to be diagnosed with ALS by 2040 (Chia et al., 2018). The precise etiology and pathogenesis of ALS remain unclear, and effective treatments are not yet available. Research from both human and animal models indicates that cellular senescence is a crucial factor in the progression of various age-related diseases (Gerenu et al., 2017; Sturmlechner et al., 2017; Xu et al., 2018; Schafer et al., 2017). Studies have also linked cellular senescence to the development of ALS (Pandya and Patani, 2020). Transcriptomic analysis of the spinal cord in hSOD1^G93A^ mice (an established mouse model of ALS) demonstrated a connection between cellular senescence and ALS, showing that 90% of age-related spinal cord transcripts were upregulated in ALS (Herskovits et al., 2018). The DNA damage response (DDR) is strongly linked to cellular senescence and can induce senescence in cells (Kang et al., 2015; Jurk et al., 2012). Our previous studies have shown that the DDR remains persistently activated in motor neurons of the hSOD1^G93A^ mice (Wang et al., 2019; Yang et al., 2020).

These findings strongly suggest that cellular senescence mediates the neuropathophysiology of ALS. Compared with other aging-related diseases, the evidence linking cellular senescence to ALS is less established. In this review, we examine the potential roles of cellular senescence in ALS, focusing on different cell types such as microglia, astrocytes, and motor neurons. Finally, we discuss the significance of senescent cell clearance for ALS treatment.

## 2 Aging of microglia

Recent studies have reported that aging microglia represent a novel therapeutic target for neurodegenerative diseases (Shahidehpour et al., 2024; Antignano et al., 2023). Research involving spatiotemporal RNA sequencing of the mouse brain identified accelerated senescence of microglial cells, particularly in white matter, and suggested that microglia may be the most prone to senescence among brain cells (Hahn et al., 2023). To date, no studies have elucidated why microglia in the white matter are particularly susceptible to aging. The threshold theory of senescent cell accumulation (Chaib et al., 2022) posits that once the quantity of senescent cells in the body exceeds a certain threshold, the immune system and other organs become more susceptible to aging-related diseases (Karin et al., 2019). Although microglia constitute only 5–10% of the total brain cells, the accumulation of senescent microglia may initiate paracrine senescence in surrounding neurons and glial cells through the senescence-associated secretory phenotype (SASP), ultimately contributing to brain-wide aging (Ng et al., 2023). Notably, an analysis of SASP shows that many of its components are biomarkers linked to neuroinflammation, most of which are substances extensively released by microglia (Matsudaira et al., 2023). Furthermore, senescent microglia exhibit altered mTOR signaling and increased oxidative stress (Palmer and Ousman, 2018). The accumulation of senescent microglia impairs their immune roles and communication with other brain cells, potentially playing a role in the initiation and advancement of neurodegenerative disorders (Angelova and Brown, 2019).

Microglia play a critical role in neuroinflammation associated with ALS (Frakes et al., 2014). Neuroinflammation involves the activation of microglia, which can polarize into either the M1 pro-inflammatory phenotype or the M2 anti-inflammatory phenotype in response to different microenvironmental stimuli (Guo et al., 2022). Thus, the role of microglia in neurodegenerative diseases is a double-edged sword. Neuroinflammation may represent a secondary consequence of cellular senescence and also act as a key contributor to brain aging (Rim et al., 2024). Studies have shown that aging of CD4 T cells intensifies neuroinflammation in a late-onset ALS mouse model. Alterations in CD4 T-cell subsets associated with aging were detected, resulting in a higher proportion of effector T cells in the spleen of SOD1^G37R^ mice (Zaccai et al., 2024). Microglia isolated from symptomatic hSOD1^G93A^ mice exhibit characteristics of the SASP, including elevated *β*-galactosidase activity, increased levels of p16, p53, MMP-1, and nitrotyrosine (Trias et al., 2019). This suggests that microglia senescence may be involved in ALS pathology. However, the mechanisms underlying the involvement of microglia senescence in ALS remain unclear. Genotoxic stress-induced senescent microglia may represent the outcome of microglial activation (Spittau, 2017).

## 3 Aging of astrocytes

Astrocytes, the predominant glial cells in the central nervous system, are essential for a range of biological functions, including neurotransmitter cycling, synaptogenesis and synaptic elimination, maintenance of the blood–brain barrier, and support for neuronal survival (Vasile et al., 2017). Astrocytes can be classified into two groups based on their response to injury mechanisms. Astrocytes exposed to inflammatory stimuli adopt the A1 phenotype, characterized by the upregulation of genes involved in synapse elimination. Conversely, astrocytes subjected to ischemia adopt the A2 phenotype, marked by the upregulation of genes that promote neurotrophy, repair, and survival (Liddelow and Barres, 2017). Levels of the astrocyte marker glial fibrillary acidic protein (GFAP) are markedly increased in the aging brain, reflecting astrocyte activation and gliosis in the context of neurodegeneration (Verkerke et al., 2021). Studies report that astrocytes in ALS exhibit an A1 reactive phenotype (Clarke et al., 2018), and in aged mice, astrocytes upregulate more A1 reactive genes compared to A2 reactive genes, suggesting that aging is linked to the more harmful A1 astrocyte phenotype (Clarke et al., 2018). Impaired astrocyte function, resulting in the continuous secretion of pro-inflammatory factors like IL-8, IL-1β, IL-6, IL-18, and TNF-*α*, has been associated with aging and age-related neurodegenerative disorders (Li et al., 2023). Transcriptomic analysis of multiple brain regions in aged mice reveals a downregulation of cholesterol synthesis in aged astrocytes. Cholesterol is a critical component for presynaptic vesicle formation, suggesting that senescent astrocytes may disrupt neuronal synaptic function and predispose to synaptic elimination (Boisvert et al., 2018).

Astrocytes have emerged as critical contributors to ALS pathology through non-cell-autonomous mechanisms, including gain of toxic functions (Nagai et al., 2007) and loss of neuronal support (Tyzack et al., 2017). Growing evidence indicates that astrocyte senescence contributes to the progression of ALS. Δ133p53, a human p53 isoform, is upregulated under low-level ROS stress to support cell survival and delay cellular senescence through upregulating antioxidant genes. Research has shown that Δ133p53 expression is reduced in the brain tissues of patients with ALS (Zhao et al., 2021). Astrocytes derived from iPSCs of patients with sporadic ALS exhibit significantly increased levels of senescence-associated markers (Birger et al., 2019). The brains of ALS patients also exhibit an increased number of senescent astrocytes (Turnquist et al., 2016). Studies have found that glial cells, primarily astrocytes, in the frontal association cortex (FACx) of ALS patients exhibit increased expression of p16 and p21, suggesting that glial cells may contribute to the induction of cellular senescence (Vazquez-Villasenor et al., 2020). Astrocytes exhibit an accelerated onset of senescence in ALS models, followed by a reduced capacity to support motor neurons (Das and Svendsen, 2015). There are numerous similarities between SOD1-overexpressing astrocytes at 150 days of end-stage and wild-type aged astrocytes at 300 days. This reveals striking similarities between ALS and wild-type aging samples (Das and Svendsen, 2015).

Oxidative stress is pivotal in ALS pathogenesis, and the increased vulnerability of senescent astrocytes to oxidative stress may be a key factor in disease progression (Barber and Shaw, 2010). Conversely, oxidative stress, including exposure to hydrogen peroxide, induces a senescent phenotype in astrocytes (Bitto et al., 2010). The interactions between astrocytes and surrounding cells are disrupted during aging, leading to the impairment of neighboring cell types through non-cell-autonomous mechanisms (Lana et al., 2019). The onset of a senescent phenotype in astrocytes can lead to various functional impairments, ultimately compromising their ability to support both themselves and neurons in the context of aging and age-related disease (Bitto et al., 2010). Additionally, astrocytes interact with microglia, the CNS immune cells, influencing their branching and distribution (Lana et al., 2019). However, during aging, these direct interactions are impaired, disrupting microglial morphology and their phagocytic capacity. This dysfunction ultimately leads to the accumulation of toxic pro-inflammatory cellular debris within the central nervous system.

Astrocytes are numerous and functionally diverse, and they undergo significant age-related changes that affect their interactions with neighboring cells, such as neurons and microglia. These alterations ultimately make the central nervous system more susceptible to age-related pathologies and neurodegenerative diseases. If astrocytes in ALS represent a pathological acceleration of the normal aging process, a deeper understanding of astrocyte senescence will provide valuable insights into the pathophysiology of ALS.

## 4 Aging of motor neurons

The aging of astrocytes and microglia has been documented, and they may disrupt glia–neuron interactions through the SASP, contributing to age-related brain pathologies (Bhat et al., 2012; Safaiyan et al., 2016; Crowe et al., 2016). Neurons are terminally differentiated cells that have left the cell cycle and ceased to proliferate. As a result, they do not undergo typical cellular senescence (Pandya and Patani, 2020). Interestingly, research shows that post-mitotic neurons can also exhibit senescence-associated alterations (Jurk et al., 2012; Piechota et al., 2016). This results in the emergence of senescence-associated neuronal traits, which could expedite neuronal dysfunction and promote the progression of neurodegenerative disorders (Vazquez-Villasenor et al., 2020). Recent studies suggest that neurons experience senescence-like changes when exposed to stress (von Zglinicki et al., 2021; Ohashi et al., 2018), and the senescence of normally aging neurons may impair their viability and increase their vulnerability to additional damage (Pandya and Patani, 2021). Elevated activity of the cellular senescence marker *β*-galactosidase has been observed in hippocampal and cerebellar granule neurons of aged rats (Geng et al., 2010; Bhanu et al., 2010). Additionally, SASP-related genes are induced, and β-galactosidase activity is elevated in neurons (Ohashi et al., 2018). Lipofuscin aggregates, which are rich in lipids, metals, and misfolded proteins, accumulate in neurons and other post-mitotic, non-proliferative cell types during normal aging (Moreno-Garcia et al., 2018). Furthermore, studies have suggested that the senescence of dopaminergic neurons plays a role in the pathogenesis of Parkinson’s disease (PD; Riessland et al., 2019). Distinct subtypes of neuronal senescence have also been identified in Alzheimer’s disease (AD; Musi et al., 2018; Dehkordi et al., 2021).

In motor neurons of ALS, the expression of p21, a cellular senescence marker, is increased (Vazquez-Villasenor et al., 2020). Neurons derived from induced pluripotent stem cells (iPSCs) of C9orf72^+^ ALS patients show a significant upregulation of senescence-associated genes (Porterfield et al., 2020). Although not neurotoxic in itself, the ectopic expression of Nav1.8 during aging can make energy-demanding motor neurons more susceptible to neurodegeneration and neuronal pathological progression (Moldovan et al., 2016). The upregulation of matrix metalloproteinases (MMP) during aging may have particular significance in ALS, as overexpression of TDP-43 in neurons accelerates the accumulation of dMMP1 (Azpurua et al., 2018). Both upper and lower motor neurons often exhibit hyperexcitability in the hSOD1^G93A^ mouse and ALS patients (Wainger et al., 2014; Tankisi et al., 2021). Excitotoxicity is characterized by the overactivation of glutamate receptors, leading to neuronal injury or cell death (Dong et al., 2009). Electrophysiological studies in ALS patients have revealed irregularities in sodium and potassium currents, indicating age-associated alterations in membrane depolarization and excitability of motor axons (Bae et al., 2008). Neurons depend on the error-prone non-homologous end joining (NHEJ) pathway for repairing DNA double-strand breaks (DSBs), and they lack the ability to mitigate the consequences of DNA repair mistakes during cell division. As a result, they are especially susceptible to DNA damage and aging (Wang et al., 2021). Research indicates that factors such as high energy demands, elevated DNA damage, the length of axons, error-prone DNA repair processes, increased susceptibility to excitotoxicity, neuronal senescence, and variability in the vulnerability of different motor neuron subtypes may collectively make them more susceptible to aging-related damage (Arnold and Clark, 2019).

**Figure 2:**
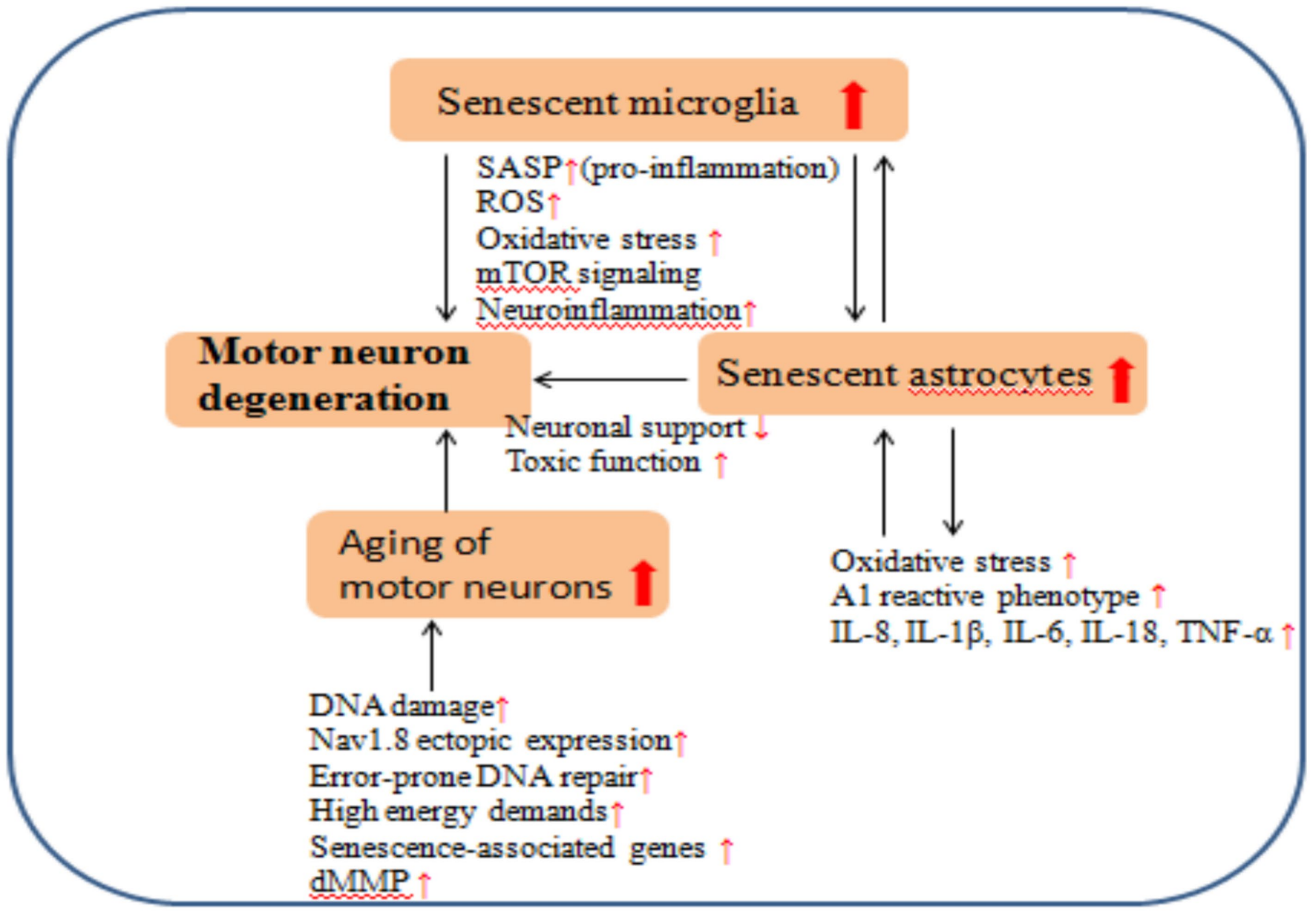
The aging of microglia, astrocytes, and motor neurons contributes to the degeneration of motor neurons in ALS.

Neuronal senescence may therefore represent a critical mechanism underlying the pathogenesis of ALS. Some studies have indicated that as the number of aging motor neurons decreases, the remaining aging motor neurons may be under higher stress (Pandya and Patani, 2020). Normal aging is accompanied by the loss of synaptic input in alpha motor neurons, a shared pathological hallmark with ALS, and leads to mitochondrial dysfunction in motor neurons. Furthermore, the accumulation of DNA damage during the normal aging may be an important risk factor for neurodegenerative diseases and ALS (Ma and Farny, 2023). Therefore, it is possible that normal aging may be a prerequisite for the degeneration of motor neurons in ALS, and aging may make this system vulnerable to the subsequent disease-specific mechanisms of ALS, although further research is needed to clearly solve this problem. Normal aging impacts the number, structure, and functional capacity of motor neurons, suggesting that age-associated changes may contribute significantly to neurodegenerative disorders affecting motor neurons, like ALS (Figure 2).

## 5 Anti-senescence therapies

Eliminating senescent cells has been reported to alleviate age-related brain diseases (Sikora et al., 2019). In paraquat-induced PD mice, the removal of senescent astrocytes reduced the loss of dopaminergic neurons and improved motor function (Chinta et al., 2018). Similarly, clearing senescent cells has been shown to mitigate chemotherapy-induced peripheral neuropathy (Acklin et al., 2020). Numerous therapeutic studies in animal models have demonstrated the feasibility of delaying aging to extend both lifespan and healthspan (Partridge et al., 2020). In AP20187 transgenic mouse models, systemic clearance of p16-positive senescent cells specifically eliminated senescent microglia and reversed cognitive decline (Zhang et al., 2022). In AD, the removal of senescent cells reduced neuroinflammation, amyloid-*β* plaque levels, and cognitive deficits (Zhang et al., 2019).

Anti-senescence drugs are compounds designed to target and eliminate senescent cells, thereby reducing the burden of these cells and extending organismal lifespan (Rim et al., 2024). Senolytic drugs can selectively target key proteins and apoptotic signaling molecules, such as PI3K/Akt and mTOR, effectively eliminating senescent cells and diminishing the SASP along with its associated consequences (Dashtmian et al., 2024). For instance, the administration of dasatinib combined with quercetin, where quercetin is a repurposed drug used as a senolytic agent, has been demonstrated to decrease senescent cell accumulation in human adipose tissue and reduce inflammation (Xu et al., 2018). Dasatinib, a broad-spectrum tyrosine kinase inhibitor targeting SRC family members, induces apoptosis of senescent cells by activating caspase-mediated pathways (caspase-3, −7, and −9; Schade et al., 2008). At the cellular level, mTOR inhibition has been shown to reverse senescence traits, including increased granularity, enlarged cell size, β-galactosidase activity, and the spindle-like morphology of fibroblasts (Walters et al., 2016). In a randomized, double-blind, placebo-controlled clinical trial for ALS, rapamycin was found to be safe and well-tolerated. However, further studies are needed to clarify its clinical and biological impacts in ALS (Mandrioli et al., 2023). Furthermore, some anti-senescence drugs that inhibit HSP90 show limited efficacy in preventing disease progression and may even counterproductively endanger degenerating motor neurons (Maximova et al., 2021). Therefore, it is still necessary to further determine the effective targets for the treatment of ALS in order to maximize the therapeutic effect.

During normal aging, cellular function gradually declines, but in ALS, this decline is accelerated. In transgenic hSOD1^G93A^ mice, administration of NMN (a CD38 inhibitor) and NR (a PARP inhibitor) delays aging, promotes stem cell renewal, and extends lifespan (Zhang et al., 2016). In hSOD1^G93A^ mice, resveratrol (an anti-aging compound) prevents motor neuron loss, alleviates muscle atrophy, improves muscle mitochondrial function, and extends survival (Mancuso et al., 2014). *In vitro* experiments have shown that inhibiting astrocyte senescence reduces the SASP and prevents astrocyte-mediated neurotoxicity (Ungerleider et al., 2021). Therefore, clearing senescent cells or restoring their normal function may be a potential strategy for treating ALS (Maximova et al., 2021).

While preclinical studies showed promising outcomes, clinical translation requires rigorous investigation. Clinical trials must evaluate the efficacy and safety of senolytic drugs in ALS patients, while elucidating how senescence contributes to disease pathology. Identifying senolytics with optimal specificity and safety profiles is essential, particularly those targeting ALS-relevant senescent cells. Large longitudinal studies will determine long-term therapeutic effects. Overcoming these challenges demands interdisciplinary collaboration to develop safe, effective ALS senotherapies.

## 6 Limitation and future directions

While cellular senescence is a key factor in age-related disorders, such as ALS, the exact triggers of senescence and the mechanisms through which senescent cells drive aging and related diseases are still not fully understood. A major challenge in this area is the detection of senescent cells *in vivo* and in postmortem tissues, emphasizing the necessity for more precise and sensitive techniques to identify these cells in living organisms. Tools for identifying and tracking senescent cells in vivo have been developed and investigated in tumors, such as the senescent-specific PET probe FPyGal, cell-free DNA (cfDNA) analysis, and fine-tuned nanoparticles for recognizing senescent cells. These advancements provide novel avenues for monitoring senescent cells in vivo in ALS. This review examines the potential contributions of cell type-specific senescence to ALS. Promising outcomes from both animal research and clinical trials support the efficacy of anti-senescence treatments. In models of several aging-related diseases, the pharmacological or genetic elimination of senescent cells has been shown to decelerate aging and mitigate senescence-associated pathophysiological changes. A deeper understanding of the mechanisms driving cellular senescence and the role of senescent cells in ALS pathogenesis could open up new avenues for therapeutic interventions. Additionally, identifying the senescence phenotypes of motor neurons in ALS patients remains a crucial focus of research, especially exploring reliable biomarkers for the senescence of motor neurons. Targeting motor neuron senescence could potentially slow disease progression in its early stages and extend the lifespan of individuals with ALS.
